# Using information technology to optimize the identification process for outpatients having blood drawn and improve patient satisfaction

**DOI:** 10.1186/s12911-022-01799-5

**Published:** 2022-03-10

**Authors:** Li-Feng Wu, Guo-Hua Zhuang, Qi-Lei Hu, Liang Zhang, Zhang-Mei Luo, Yin-Jiang Lv, Jian Tang

**Affiliations:** 1 Department of Clinical Laboratory, First People’s Hospital of Linping District, Hangzhou, Hangzhou, 311100 Zhejiang China; 2 Quality Management Section, First People’s Hospital of Linping District, Hangzhou, No. 369 of Yingbin Road, Linping District, Hangzhou, 311100 Zhejiang China

**Keywords:** Information technology, Patient identification, Process optimization, Patient satisfaction

## Abstract

**Background:**

This study explored the application effect of information technology in optimizing the patient identification process.

**Methods:**

The method for optimizing the identification process involved in drawing blood among outpatients using information technology was executed from July 2020. In this paper, 959 patients who had blood drawn from January to June 2020 were included as the pre-optimization group, and 1011 patients who had blood drawn from July to December 2019 were included as the post-optimization group. The correct rate of patient identification, waiting time, and patient satisfaction before and after the optimization were statistically analyzed. The changes in these three indexes before and after the optimization implementation, as well as the application effects, were compared.

**Results:**

The correct rate of patient identification after optimization (99.80%) was higher than before optimization (98.02%) (X^2^ = 13.120; *P* < 0.001), and the waiting time for having blood drawn was also significantly shortened (t = 8.046; *P* < 0.001). The satisfaction of patients was also significantly improved (X^2^ = 20.973; *P* < 0.001).

**Conclusions:**

By combining information technology with the characteristics of blood collection in our hospital, using the call system to obtain patient information, then scan the QR code of the guide sheet for automatic verification, and finally manually reconfirm patient information, which can significantly reduce the occurrence of identification errors, improve work efficiency and improve patients' satisfaction.

## Background

Patient identification refers to the process in which medical personnel check and verify the identity of patients within a range of medical activities to ensure that the correct treatment and examination are effected for each patient. Correct patient identification is the foundation for ensuring patient safety [1, 2]. In the ten safety objectives for patients issued by the Chinese Hospital Association in 2019, the strict implementation of a checking system and the application of barcode scanning technology are highlighted and encouraged to ensure that correct patient identification takes precedence [3]. Patient identity security and satisfaction are the focus of the Joint Commission on Accreditation of Healthcare Organizations’ standards, of which more than 50% are related to these two factors [4].

Correct patient identification is also the first step for medical staff in conducting any treatment. A UK study showed that, between 2006 and 2008, there were 1,309 incidents related to patient misidentification, 97% of which occurred in hospitals [5]. Further studies in the US concluded that laboratory-related work was the most common cause of identification errors (31%), followed by medication errors (22%), invasive procedures or surgery on the wrong patient (19%), and diagnostic imaging errors (17%) [6]. The Joint Commission reported a 12.3% incidence of adverse events related to patient identification between 2014 and 2017 [7], and in China, adverse events (such as surgical errors and infusion errors) caused by patient misidentification have been found to occur at all levels of medical care [8]. As identification errors can lead to misdiagnosis and mistreatment and cause a significant waste of resources, patient identification management is particularly important.

Correct patient identification is the first step in outpatient blood collection, where it serves as a cornerstone for the accuracy of related tests and provides a fundamental guarantee of safe medical treatment [9, 10]. At present, identification errors in outpatient blood collection are mostly caused by manual verification systems that do not use at least two methods for verification [11]. However, information technology can now be widely used in patient identification, and patient identification tools and technologies are showing a diversified trend, with bar codes, radio frequency identification tags, and biometric identification (such as face and fingerprint recognition) being gradually introduced into clinical practice. Information technology applied to the management of blood collection in hospitals has been found to achieve remarkable results [12, 13], so it is clear that it is an important measure for improving the accuracy of patient identification in outpatient blood collection, thereby ensuring the correct implementation of various diagnoses and treatment operations. Improving the accuracy of outpatient identification ensures the safety of patients and improves their medical experience and satisfaction. However, the implementation, transformation, and optimization of information technology should be based on the existing processes of each hospital.

Reducing errors caused by the misidentification of patients has always been a problem in outpatient blood collection processes, and it is the primary complaint among patients in diagnosis and treatment disputes [14, 15]. To avoid the occurrence of patient identification errors, the present study adopted information technology to optimize the patient identification process, thereby ensuring the accuracy of patient identity checks and improving patient satisfaction.

## Methods

### General information

In July 2020 in First People's Hospital of Linping District, Hangzhou, the patient identification process within the outpatient blood-collection window was optimized. There were 132,676 patients before optimization and 153,283 patients after optimization. Patients who needed to have blood drawn between January 2020 and June 2020 were enrolled in the study and designated as the pre-optimization group, while patients requiring the same process between July 2020 and December 2020 were designated as the post-optimization group. Based on the Joint Commission’s international hospital evaluation standards (5th edition), 128 cases were randomly selected each month before and after optimization; combined with self-examination (180 cases before optimization, 240 cases after optimization) and related petition events (11 cases before optimization, three cases after optimization), there were 959 cases before optimization and 1,011 cases after optimization.

### Data and methods

#### Materials

A queue management system and queuing machine were implemented using the Laboratory Information Management System (LIS) of Hangzhou Lianzhong Medical Science Co., Ltd. (China). Software engineers from the company repaired and optimized the patient identification process for outpatient blood collection by modifying internal procedures.

#### The pre-optimization process of patient identification in outpatient blood collection and its associated problems

Prior to optimization, the process of patient identification in outpatient blood collection was as follows. Outpatient physicians issued test requests based on medical orders, after which the patient took a numbered ticket from a dispenser in the outpatient division for blood collection. The blood-collection staff called out the numbers, scanned/imported the patient’s outpatient number to query the test items, asked the patient their name, printed the barcode identifying the required test items, and drew samples of the patient’s blood.

This process was accompanied by three significant problems: (1) the queuing machine only had a queue number, so the number of multiple taking was different and there was no restriction on test items; (2) there was no patient information in the machine, so the blood-collection staff needed to scan a code to extract the patient’s information, and this information could only be verified by asking the patient; (3) there was no reminder of priority treatment on the receipt.

#### Solutions identified and implemented post-optimization

Through practice, the problems associated with patient identification in the outpatient blood-collection process were identified and optimized. (1) When the patient took a number from the queuing machine, a limitation was added to the inspection items; this number could not be obtained without payment or after blood collection, and a relevant prompt was provided by the system. In addition, the patient’s name was added to the number retrieval form (part of the patient’s name was removed to ensure privacy protection). If the patient did not have blood drawn after taking a number, the number taken again was the same as the first number. (2) As the blood-collection staff called out each number, they could directly read the patient’s test items using the information ascribed to that number and were able to query this information of the two patients with the prior numbers. (3) When recording the patient’s name, the outpatient number was scanned/input into the LIS. The system automatically checked the patient’s information (name, age, outpatient number, etc.) and test items captured by the calling system; if the details were identical, the verification was complete, but if it was not, the system automatically reminded staff that manual verification was required before the next procedure. In addition, the patient’s information was reserved for a secondary check. (4) For patients requiring priority treatment, warm tips were added; these patients were given priority for having their blood drawn in the preferential treatment window.

### Observation indexes

The observation indexes before and after optimization were compared. The rate of correct patient identification (%) was calculated as: number of patients correctly identified / total number of patients × 100. The waiting time for outpatient blood collection was identified as the period from when the patient took a ticket number to the start of blood collection. This time period was exported from the hospital’s information system for statistical analysis. The statistical scope included all of the study subjects. The patient satisfaction survey was based on the follow-up results of patients, which were obtained from the hospital’s quality management department.

### Statistical analysis

SPSS 22.0 software was used for data processing. A Kolmogorov–Smirnov test was employed to analyze the distribution of data. Normally distributed measurement data were expressed as the mean ± standard deviation ($\overline{x}$ ± SD) and were compared between the two groups using an independent two-sample *t*-test. Percentages were compared using a χ^2^ test. *P* < 0.05 was considered statistically significant.

## Results

### Comparison of the rate of correct patient identification before and after optimization

There were 132,676 patients before optimization and 153,286 patients after optimization. According to the requirements of the sampling optimization of 959 patients and 1,011 patients after optimization, the number of correctly identified patients before optimization was 940, and the number of correctly identified patients after optimization was 1,009. Statistical analysis showed that the rate of correct identification before optimization (98.02%) was significantly lower than that after optimization (99.80%), showing that the accuracy of patient identification significantly improved after the implementation of the new patient identification process (χ^2^ = 13.120, *P* < 0.001) (see Table 1).

**Table 1:** Comparison of correct rate before and after optimization of patient identification process

Group	Total cases (n)	Number of correctly identified cases (n)	Correct rate of identification (%)
Pre optimization group	959	940	98.02
Post optimization group	1011	1009	99.80
χ^2^	–	–	13.120
*P*	–	–	< 0.001

### Comparison of waiting times for blood-drawing procedures before and after optimization

A summary analysis of the waiting times of 959 patients before optimization and 1,011 patients after optimization showed that, after optimization, a waiting time of 0–30 min accounted for 89.81% of cases—29.85% more than before optimization. The proportion of patients waiting for 31–60 min also decreased from 36.81% before optimization to 10% after optimization, and the number of patients waiting for more than 60 min decreased from 3.23% before optimization to 0.19%. The mean waiting time was also significantly shortened after optimization (*t* = 8.046, *P* < 0.001) (see Table 2).

**Table 2:** Comparison of waiting time for blood drawing between the two groups

Group	Total cases (n)	0–30 min (n/%)	31–60 min (n/%)	> 60 min (n/%)	Average waiting time ($\overline{x}$ ± SD) min
Pre optimization group	959	575/59.96	353/36.81	31/3.23	27.36 ± 11.69
Post optimization group	1011	908/89.81	101/10.00	2/0.19	18.95 ± 7.28
χ^2^/*t*	–	χ^2^ = 22.315	χ^2^ = 15.635	χ^2^ = 18.029	*t* = 8.046
*P*	–	< 0.001	< 0.001	< 0.001	< 0.001

### Comparison of patient satisfaction before and after optimization

Patient satisfaction was defined on a scale of “very satisfied”, “satisfied”, “general”, and “dissatisfied”. A summary analysis of the satisfaction of 959 patients before optimization and 1,011 patients after optimization showed that, after optimization, the proportion of very satisfied patients was 69.14–11.16% higher than before optimization—and that the proportion of satisfied patients decreased from 36.40% to 29.17% after optimization. The proportion of general and dissatisfied patients also decreased significantly after optimization, and the satisfied, general, and dissatisfied patients from before optimization became very satisfied patients after optimization. This shows that optimizing the patient identification process significantly improved patient satisfaction rate (χ^2^ = 20.973; *P* < 0.001); in particular, the rate of very satisfied patients improved significantly (see Table 3).

**Table 3:** Comparison of patients' satisfaction between the two groups

Group	Total cases (n)	Very satisfied (n/%)	Satisfied (n/%)	General (n/%)	Unsatisfied (n/%)	Satisfaction rate
Pre optimization group	959	556/57.98	349/36.40	23/2.40	31/3.23	94.37
Post optimization group	1011	699/69.14	295/29.17	8/0.79	9/0.89	98.32
χ^2^	–	16.234	8.517	12.054	13.409	20.973
*P*	–	< 0.001	0.032	< 0.001	< 0.001	< 0.001

## Discussion

Paying more attention to the treatment of diseases and patients and emphasizing medical and patient safety require changes in management concepts; these changes denote not only the enhancement of quality management but also the concrete embodiment of patient-centered concepts [16]. Patient safety is vital in all aspects of medical treatment. All standards represent general requirements, however, and it can be difficult to provide specific rules for individual clinical links [17].

The identification of outpatients in the context of having blood drawn serves as a basis for laboratory work, so it is vital that it is conducted accurately. However, factors such as staff attitudes and energy levels, in addition to other subjective and objective reasons, may lead to mistakes being made. These errors can be reduced (or even eliminated) only through careful system construction and process optimization. To ensure patient safety, an approach for continuously improving patient satisfaction is also an important factor for evaluating diagnosis and treatment levels. It is vital for ensuring the safety, quality, and quantity of patients, improving patient identification, reducing waiting times for outpatients having blood drawn, and improving patient satisfaction.

The present study found that using information technology to optimize the identification process for patients having blood drawn improved the rate of correct identification. Furthermore, it reduced the waiting time for patients even under conditions of increased patient numbers, minimizing work intensity for blood-collection staff and improving the experience for patients. Following the process optimization, identification errors occurred for only two patients, both of which arose as a result of patients having different names with the same pronunciation and due to staff (who had recently participated in pre-employment training before immediately taking up their medical role) still checking the patient information according to the pre-optimization process; these errors were identified and corrected in time during the instructors’ inspections. By summarizing the experience and lessons of this optimization, continuous improvement can be effected through follow-up personnel management (i.e., after initial training, medical staff must continue to be trained and monitored for a certain time before working independently).

After optimizing the identification process for patients having blood drawn, the waiting time for patients was significantly reduced, with the procedure being effected within 30 min in almost all cases; in only two cases, the waiting time was over 60, and tracking analysis found that this was caused by a fault in the ticket-dispensing machine. This underscores the need not only for process optimization but also for the regular maintenance of related hardware.

Following optimization of the identification process, the patient satisfaction rate was also significantly improved, and the rate of very satisfied patients was particularly noticeable. This improvement of satisfaction was related to ensuring the safety of patients and closely linked to significantly shortened waiting times. However, the exact correlation between waiting times and patient satisfaction was not analyzed in the present study, which represents a limitation of the study that should be addressed in future research.

In the present study, the patient identification information and test items were first read by the calling system, automatically checked by scanning the QR code of the patient guide sheet, and finally supplemented by manual checking of the patient identification information, creating a simple and fast patient identification method. This sets a precedent in the use of information technology for patient identification in outpatient blood collection and automated patient identification. In addition, it shows that manual identification, which is prone to mistakes, can be replaced by a secondary method that allows staff to focus on addressing more complex problems, thereby reducing labor intensity, guiding the organic integration of artificial intelligence systems, and integrating ways to minimize patient identification errors and improve staff labor efficiency.

The results of the present study are consistent with those of Xing et al. [18]; however, the research method used in this study was simpler than the one employed by Xing et al. and can therefore be more easily popularized and applied in primary hospitals. The results are also similar to those found by Liu et al. [19], but Liu et al. that employed information technology to identify surgical patients rather than outpatients requiring blood collection.

It is important to note that, before the application of information technology to optimize the patient identification process, the existing processes of a unit must be systematically summarized to identify problems. Furthermore, the optimization process is gradual: whenever problems are found, targeted rectification is required in order to minimize errors in patient identification.

## Conclusion

With the rapid development of information technology in recent years, integrating this technology with patient identification has become a popular research direction. Information technology can realize the automation and standardization of selected links, which can reduce staff workload and improve the accuracy of results. However, this advanced technology requires stronger cooperation among staff members. Therefore, the strengthening of personnel training must remain consistent with changing technology. Ultimately, patient safety is paramount. Accordingly, manual secondary checks remain essential to processes involving patients.

By combining information technology with the characteristics of the blood-collection process in First People's Hospital of Linping District, Hangzhou by using a call system to obtain patient information, scanning a QR code on the patient guide sheet for automatic verification, and manually reconfirming patient information, the occurrence of identification errors was reduced and work efficiency and patient satisfaction was improved. The study also showed that any problems in the pre-optimization process should be systematically analyzed in order to optimize it most effectively.

## Data Availability

All data generated or analysed during this study are included in this published article.
